# Clinical benefit of antiangiogenic therapy in advanced and metastatic chondrosarcoma

**DOI:** 10.1007/s12032-017-1030-2

**Published:** 2017-08-29

**Authors:** Robin L. Jones, Daniela Katz, Elizabeth T. Loggers, Darin Davidson, Eve T. Rodler, Seth M. Pollack

**Affiliations:** 1 Fred Hutchinson Cancer Research Center, University of Washington, Seattle, WA USA; 2 Sarcoma Unit, Royal Marsden Hospital/Institute of Cancer Research, Fulham Road, London, SW3 6JJ UK; 3 Institute of Oncology, Assaf Harofeh Medical Center, Zrifin, Israel; 4 Division of Oncology, University of Washington, Seattle, WA USA; 5 Department of Orthopaedics and Sports Medicine, University of Washington, Seattle, WA USA; 6 Division of Oncology, University of California Davis, Sacramento, CA USA

**Keywords:** Chondrosarcoma, Advanced/metastatic, Systemic therapy, Antiangiogenic, Pazopanib, Ramucirumab

## Abstract

Chondrosarcoma is the most common bone sarcoma in adults. Conventional chondrosarcoma, the commonest histological subtype, is largely resistant to anthracycline-based chemotherapy. There have been anecdotal reports of durable clinical benefit with antiangiogenic agents in this disease. A retrospective search of patients treated at three sarcoma referral centers was performed to identify patients with advanced chondrosarcoma treated with antiangiogenic agents. The aim of this study was to evaluate the efficacy and safety of antiangiogenic agents in advanced chondrosarcoma. Ten patients were identified; seven with conventional, one each with clear cell, extraskeletal mesenchymal chondrosarcoma and extraskeletal myxoid chondrosarcoma. The median progression-free survival for patients with conventional and clear cell sarcoma was 22.6 months. Median overall survival has not been met. Antiangiogenic therapy was well tolerated in this series of patients. Our retrospective data suggest that antiangiogenic therapy can provide prolonged clinical benefit in advanced chondrosarcoma patients. Further prospective trials are required to precisely define the role of this class of agent in advanced chondrosarcoma.

## Introduction

Chondrosarcoma is a malignant tumor characterized by the production of cartilage matrix and displaying varied histopathology and clinical behavior [1]. Chondrosarcomas are the most common bone sarcomas in adults and following myeloma and osteosarcoma, the third most frequent primary malignant tumor of bone. There are several histological subtypes, including conventional, mesenchymal, dedifferentiated and clear cell. The conventional subtype accounts for approximately 85% of all chondrosarcomas. Two rare subtypes, extraskeletal mesenchymal and extraskeletal myxoid chondrosarcoma, do not carry typical features of chondrosarcoma. Extraskeletal mesenchymal chondrosarcoma carry mixed features of Ewing sarcoma and chondrosarcoma, while extraskeletal myxoid chondrosarcoma is a soft tissue sarcoma.

Complete surgical resection is the mainstay of management of localized disease; however, approximately 70% of patients with grade 3 tumors will develop metastatic disease. There is no standard systemic therapy for metastatic chondrosarcoma. A large retrospective study analyzed the benefit of first-line chemotherapy in 180 patients with advanced chondrosarcoma [2]. One hundred and thirteen patients had conventional chondrosarcoma (63%), 42 (23.5%) dedifferentiated, 17 (9.5%) mesenchymal and 2 (1%) clear cell. The objective response rate by RECIST was significantly different according to histological subtype; 31% for mesenchymal, 20.5% for dedifferentiated, 11.5% for conventional and 0% for clear cell sarcoma (*p* = 0.04). The median progression-free survival was 4.7 months (95% CI 3–6.4). The SARC 003 trial recruited 25 chondrosarcoma patients, and the cohort was closed due to slow accrual and low probability of achieving the target response rate of 20% [3]. Consequently, there is a clear need for more effective systemic therapy for patients with metastatic/advanced chondrosarcoma.

Angiogenesis pathways are potentially effective targets for arresting the growth and spread of chondrosarcomas [4]. Because diffusion of adequate amounts of oxygen for aerobic metabolism is limited to 200 μm, it is necessary for tumors to develop a vasculature to grow beyond several millimeters in size. Although originating from cartilaginous tissue, which is one of a handful of avascular tissues in the human body, chondrosarcomas have been shown to exhibit a microvascularity that has been associated with aggressive clinical behavior and a higher potential for metastasis [4, 5]. In 2011, a study involving 58 conventional chondrosarcomas found that microvessel densities in chondrosarcoma tumors correlate with histological grade and subsequently prognosis, suggestive of a role for neovasculature in the clinical behavior of chondrosarcoma [6]. VEGF (vascular endothelial growth factor) is essential for the neovascularization required to sustain and propagate a tumor [7]. Efforts to develop antiangiogenic therapies have produced many agents including the small molecule tyrosine kinase inhibitor, pazopanib, and full human monoclonal (IgG1) antibodies, such as bevacizumab and ramucirumab, which affect angiogenesis by binding VEGF or the VEGF receptor (VEGFR), respectively.

Pazopanib is a multitargeted tyrosine kinase inhibitor that inhibits angiogenesis pathways, namely VEGF, and has shown significant effect on tumor vascular density, viability, and volume in mice with chondrosarcoma xenografts [8]. Pazopanib has been shown to provide a benefit to nonadipocytic soft tissue sarcoma patients by extending progression-free survival by several months when compared to a placebo [9]. Ramucirumab is a monoclonal antibody against the extracellular domain of VEGFR2 [10] and has shown significant antitumor activity in patients with advanced solid tumors in recent clinical trials [11].

Due to the preclinical data suggesting potential benefit of antiangiogenic therapy in chondrosarcoma, a number of patients with metastatic chondrosarcoma have been treated with such agents within clinical trials or off trial as compassionate treatment at our institutions. Due to the limited published data on effective systemic therapy in this disease, the aim of our study was to report the clinical activity and safety of antiangiogenic agents in advanced chondrosarcoma.

## Materials and methods

Prior to commencing the study, institutional review board approval was obtained from the Fred Hutchinson Cancer Research Center, Royal Marsden Hospital and the Hadassah-Hebrew University Medical Center. Approval included authorization to perform a retrospective search of the electronic patient record for each patient. A retrospective search was performed at the University of Washington, the Royal Marsden Hospital and the Hadassah-Hebrew University Medical Center, to identify chondrosarcoma patients treated with antiangiogenic agents between 2010 and 2016. Date of diagnosis, age, gender, surgery, radiation and prior systemic therapy were obtained from the electronic patient record. Data regarding toxicity were also obtained from the electronic patient record. Toxicity was graded according to the National Cancer Institute Common Toxicity Criteria grading system and recorded at each clinic visit.

Pazopanib was prescribed at 800 mg per day. Ramucirumab was administered as per clinical trial protocol: 10 mg per kg intravenously over 60 min every 3 weeks. Premedication with 25–50 mg of intravenous diphenhydramine was a requirement before each dose of ramucirumab. Dose reductions were performed as per institutional guidelines or clinical trial protocol.

All patients had progressive disease prior to commencing antiangiogenic therapy and underwent a baseline pre-treatment scan. Response to treatment was assessed using RECIST (Response Evaluation Criteria in Solid Tumors) 1.1. Re-staging scans were performed every 6–8 weeks or according to clinical trial protocol or standard guidelines. The diagnosis of chondrosarcoma was confirmed in all cases by experienced sarcoma pathologists. Safety data were recorded and retrieved from the patient medical record.

Descriptive statistics were employed. Progression-free and overall survival were calculated using the Kaplan–Meier method.

## Results

Ten chondrosarcoma patients treated with antiangiogenic therapy were identified. The median age of this cohort was 53 years (range 34–72). One patient each had extraskeletal mesenchymal chondrosarcoma, extraskeletal myxoid chondrosarcoma and clear cell sarcoma. All other patients had conventional chondrosarcoma. All but one of these patients had undergone prior surgery and six of the ten had received prior systemic therapy. The cohort included a variety of anatomic primary sites including upper and lower extremities as well as chest wall and pelvis. The clinical characteristics of these patients are displayed in Table 1. Eight patients were treated at the Fred Hutchinson Cancer Research Center/University of Washington. One patient was treated at the Royal Marsden and one at the Hadassah-Hebrew University Medical Center [12]. Both patients which course is described in the results section provided consent for publication of the study.

**Table 1:** Patient clinical characteristics

Patient	Gender	Age at diagnosis (years)	Subtype	Primary site	Time to metastatic disease (months)	Metastatic sites	Prior surgery	Prior therapy
1	F	34	Conventional	Lung	0.1	Skull, hepatic, kidney, soft tissue	Yes	Trial
2	F	50	Conventional	Femur	2.6	Lung	Yes	Trial
3	M	60	Conventional	Chest wall	0.3	Lung	Yes	None
4	M	61	Conventional	Pelvis	14.0	None	Yes	Trial
5	M	40	Conventional	Femur	4.3	Lung, spleen	Yes	Gemcitabine/docetaxel Trial
6	F	42	ESMC	Limb	4.0	Lung, soft tissue	Yes	Doxorubicin/ifosfamide Gemcitabine/docetaxel
7	M	72	EMC	Chest wall	0	Lung	Yes	None
8	M	57	Clear Cell	Limb	3.1	Lung	None	None
9	F	53	Conventional	Spine	Residual disease	None	Yes	None
10^a^	M	56	Conventional	Limb	47	Mediastinum	Yes	Gemcitabine/docetaxel

^a^Reference [12] presents a shorter follow up of this patient

### Safety

All patients tolerated treatment well and there was one grade 3 toxicity and no patient discontinued therapy due to toxicity. One patient developed melena approximately 8 months after initiating therapy. The hemoglobin level decreased to 7.6 (Grade 3). Endoscopy and colonoscopy did not demonstrate a source of bleeding. However, due to microcytic anemia, pazopanib was stopped 8 months after initiating treatment, and the hemoglobin level subsequently stabilized. Hypertension and fatigue were the most common toxicities in this series (Table 2). Five patients were commenced on anti-hypertensive therapy. There were no treatment related deaths. Three patients died of progressive disease.

**Table 2:** Antiangiogenic therapy, toxicity and response

Patient	Agent	Toxicity	Best response	Time to progression (months)
1	Pazopanib	Nausea, fatigue, dry skin, vision changes, tumor pain	Stable disease	>13
2	Pazopanib	Hypertension, tumor pain	Progressive disease	2
3	Pazopanib	Hypertension	Progressive disease	1
4	Ramucirumab	Nausea, leukopenia	Stable disease	5
5	Ramucirumab	Fatigue, nausea, tumor pain	Stable disease	23
6	Pazopanib	Fatigue, hypertension, diarrhea, nausea, myalgia, tumor pain	Stable disease	>26
7	Pazopanib	Fatigue	Stable disease	8
8	Pazopanib	None	Stable disease	>6
9	Pazopanib	Mucositis	Stable disease	>6
10^a^	Pazopanib + sirolimus	None	Stable disease	8

^a^Reference [12] presents a shorter follow up of this patient

### Efficacy

**Fig. 1:**
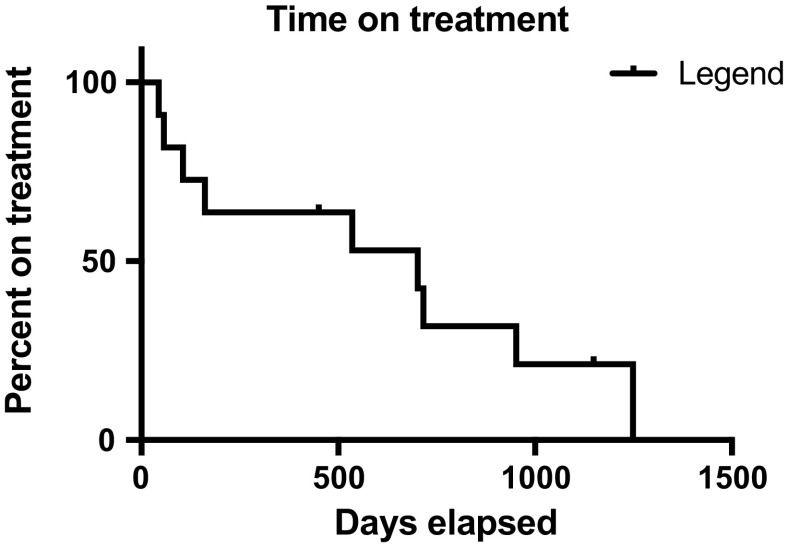
The Kaplan–Meier curve for progression-free survival for all 10 patients

**Fig. 2:**
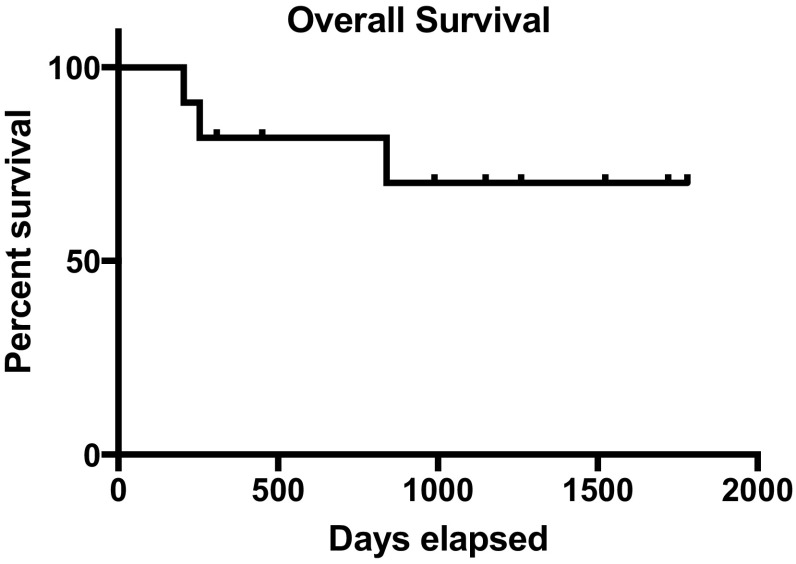
The Kaplan–Meier curve for overall survival for all 10 patients

Eight patients were treated with pazopanib and 2 with ramucirumab (within clinical trials). No RECIST partial responses were documented; however, seven patients achieved prolonged disease stabilization for over 6 months, with one patient with conventional chondrosarcoma on ramucirumab for 23 months (Table 2). The median progression-free survival for the eight patients with conventional and clear cell sarcoma was 22.6 months. The median overall survival has not been met. The Kaplan–Meier curves for progression-free and overall survival are shown in Figs. 1 and 2, respectively.

In order to better understand the clinical course of patients with advanced chondrosarcoma treated, we provide case descriptions below of the two patients with the most durable responses to antiangiogenic therapy.

One patient aged 50 years (Patient 2) developed a left distal femoral conventional chondrosarcoma 6 years following treatment (chemotherapy and radiation) for stage III Hodgkin’s disease. The grade 2 chondrosarcoma was treated with surgery. Three years following surgery he developed a local recurrence, which was resected. Seventeen months following this a metastatic lung nodule was resected and then 3 months later he underwent a left below knee amputation for a further local recurrence. Four months later he received two cycles of gemcitabine and docetaxel for progressive lung metastases. The disease progressed on gemcitabine and docetaxel, and he then entered a Phase II trial of dasatinib for 8 months. He was subsequently entered a Phase I trial of ramucirumab for 2 years with prolonged stable disease. He tolerated treatment with ramucirumab well and had an excellent quality of life. On the development of progressive disease, he was entered into a randomized Phase II trial of a smoothened inhibitor versus placebo. The disease progressed on the first re-staging CT scan.

A 57-year-old man (Patient 8) initially underwent resection of a clear cell sarcoma of the left distal femur. Two years following primary surgery he developed a local recurrence and underwent further resection, but a subsequent PET-CT 6 months later demonstrated multiple metastatic lung lesions. Repeat imaging revealed progressive disease with new lung nodules, and he was then commenced on pazopanib. He has been on pazopanib for over 2.5 years with stable disease and an excellent quality of life.

## Conclusion

Our retrospective study shows that patients with advanced/metastatic chondrosarcoma can obtain prolonged clinical benefit with antiangiogenic therapy which was well tolerated. None of the patients had a partial response, and the clinical benefit was observed in terms of prolonged stable disease in patients experiencing disease progression prior to commencing therapy. For the seven patients with conventional and one with clear cell chondrosarcoma, the median PFS of 22.6 months is promising, particularly in the context of the data reported by Italiano and colleagues of chemotherapy in chondrosarcoma with a median PFS of 4.7 months.

A number of promising preclinical studies have suggested that antiangiogenic therapy could be a potentially useful approach in chondrosarcoma [8]. Ayala et al. [5] demonstrated a correlation of pericartilage blood vessels and higher-grade chondrosarcoma tumors and showed intracartilage vessels are predominantly manifested in high-grade tumors. They also showed that VEGF expression in malignant chondrocytes is nearly exclusive to high-grade lesions.

Furumatsu et al. [7] studied the angiogenic activities of human chondrosarcoma cell line, OUMS-27, and its effect on human umbilical vein endothelial cells (HUVECS) in vitro. They demonstrated that VEGF is the principal angiogenic factor in prompting HUVEC propagation and migration and that VEGF targeted antibodies can restrict these endothelial responses by about 70%, suggesting that there is a place for a VEGF targeted therapy in the management of chondrosarcoma [7]. Furthermore, Morioka et al. [13] showed efficacy of antiangiogenic molecules in treating chondrosarcoma xenografts in mice. This study demonstrated that plasminogen-related protein B, an antiangiogenic protein expressed in normal human cartilage tissue, showed a significant effect in mitigating neoplastic growth in human chondrosarcoma xenografts in mice. Levine et al. [14] showed that antisense oligonucleotides against VEGF inhibit tumor cell proliferation in human cancer, including one patient with chondrosarcoma. Stacchiotti et al. [15] have also reported on the activity of sunitinib in extraskeletal myxoid chondrosarcoma.

Although our study is limited by small patient numbers and its retrospective nature, it does suggest that further prospective studies are required to better define the role of antiangiogenic therapy in chondrosarcoma, as the systemic options available for patients with inoperable disease are limited. There are currently studies analyzing the efficacy of pazopanib in the management of surgically unresectable or metastatic chondrosarcoma patients (NCT01330966, NCT02066285).
